# Tractography of the arcuate fasciculus in healthy right-handed and left-handed multilingual subjects and its relation to language lateralization on functional MRI

**DOI:** 10.1038/s41598-021-00490-5

**Published:** 2021-10-22

**Authors:** Sandrine Yazbek, Stephanie Hage, Iyad Mallak, Tarek Smayra

**Affiliations:** Medical School, Hotel-Dieu de France Hospital, Saint Joseph University, Boulevard Alfred Naccache, Achrafieh, PO Box 166830, Beirut, Lebanon

**Keywords:** Neuroscience, Medical research

## Abstract

Functional MRI (fMRI) enables evaluation of language cortical organization and plays a central role in surgical planning. Diffusion Tensor Imaging (DTI) or Tractography, allows evaluation of the white matter fibers involved in language. Unlike fMRI, DTI does not rely on the patient’s cooperation. In monolinguals, there is a significant correlation between the lateralization of language on fMRI and on DTI. Our objective is to delineate the arcuate fasciculus (AF) in right- and left-handed trilinguals and determine if the AF laterality on DTI is correlated to language lateralization on fMRI. 15 right and 15 left-handed trilingual volunteers underwent fMRI and DTI. Laterality Index was determined on fMRI (fMRI-LI). Mean Diffusivity, Fractional Anisotropy (FA), Number of Fibers, Fiber Length, Fiber Volume and Laterality Index (DTI-LI) of the AF were calculated on DTI. 28 of the 30 subjects presented a bilateral AF. Most subjects (52%) were found to have a bilateral language lateralization of the AF on DTI. Only 4 subjects had bilateral lateralization of language on fMRI. The right AF demonstrated lower diffusivity than the left AF in the total participants, the right-handed, and the left-handed subjects. FA, Volume and Length of the AF were not significantly different between the two hemispheres. No correlation was found between the DTI-LI of the AF and the fMRI-LI. A prominent role of the right AF and a bilateral structural organization of the AF was present in our multilingual population regardless of their handedness. While in prior studies DTI was able to determine language lateralization in monolingual subjects, this was not possible in trilingual highly educated subjects.

## Introduction

Language lateralization and localization to the left frontal hemisphere has been described by Paul Broca in 1865^1^. Broca’s observation of an exclusive left hemispheric dominance has been challenged since. Even though leftward structural and morphological asymmetries are found in the language areas of the left cerebral hemisphere^2^, right dominance of language and bilateral activation of language are present in the left-handed subjects^3^ and, less frequently, in the right-handed subjects^4^. This variation in the hemispheric dominance of language illustrates why evaluation of language lateralization is important preoperatively. Language lateralization used to be determined pre-operatively using the sodium amytal test, also called Wada test, named after Juhn A. Wada a Japanese-born neurologist^5^. With the advent of MRI, functional MRI (fMRI) has supplanted the Wada test for evaluation of language dominance. fMRI is less invasive than the Wada test, it does not rely on radiation and has good concordance with Wada results^6,7^. While fMRI studies activation and localization of the language areas in the cortex of the cerebral hemispheres, Diffusion Tractography Imaging (DTI) permits evaluation of the white matter fibers involved in language. Unlike fMRI, Tractography is a technique that does not rely on the patient's cooperation and paradigm testing during imaging. It can even be performed under sedation. DTI permits evaluation of the Arcuate Fasciculus (AF) which is the white matter tract that connects the temporal, parietal and frontal regions via one direct and two indirect trajectories between Broca’s and Wernicke’s territories^8,9^. A leftward laterality of the AF regarding Fractional Anisotropy (FA)^10^, volume^11–13^ and fiber density^14,15^ has been described. Several studies combining DTI tractography and fMRI demonstrated a significant correlation between the lateralization of language on fMRI and the lateralization of FA^16^ and volume of the AF^17^. Most of these studies do not mention whether their subjects are monolinguals or multilinguals. Differences in language lateralization and activation on fMRI in multilingual subjects compared to monolinguals have been evaluated^18–20^. When the age of acquisition of the second language is less than 6 years of age, there is recruitment of additional areas of activation on fMRI especially in the right hemisphere. This leads to a more bilateral lateralization of language in early bilinguals compared to late bilinguals or monolinguals^21–23^. Proficiency has also been demonstrated to play a major role in language activation in multilingual subjects on fMRI. The language with the least proficiency activates larger and more numerous areas in the brain on fMRI^24,25^.

Described structural differences in the white matter tracts between bilingual and monolingual subjects include higher FA mean values of the language pathways in bilingual subjects^26,27^. However, most research on the white matter has not taken advantage of the structural differences of the language white matter tract between the monolingual and the multilingual populations and their relation to language lateralization. Evaluation of the lateralization of the AF on DTI in multilinguals subjects has important implications for a better understanding of the functional and structural lateralization of the language areas of the brain as well as for the evaluation of language function of multilingual subjects in a clinical setting. To the best of our knowledge, there are no studies evaluating the correlation between the language lateralization on fMRI and the structural characteristics of the AF in a multilingual population.

Our objective is to delineate the arcuate fasciculus (AF) in right-handed and left-handed trilingual subjects and to determine the relation between the AF characteristics on DTI and the results of language lateralization on fMRI. Based on the available data in the literature in monolingual subjects, we hypothesized that most right-handed trilingual volunteers would present leftward asymmetry of the AF that would correlate with a left lateralization of language on fMRI.

## Material and methods

### Subjects

The study protocol was reviewed and approved by the Ethical Committee of St-Joseph University—Hôtel-Dieu de France hospital, and has been performed in accordance with the ethical standards laid down in the 1964 Declaration of Helsinki and its later amendments. All subjects gave written informed consent in order to participate in the study.

15 left-handed and 15 right-handed healthy trilingual individuals were included in this prospective study. Handedness of the subjects was determined by the Edinburgh Handedness Inventory^28^ on the basis of which a Laterality Quotient (EHI LQ) was calculated. Left handedness was determined with an EHI LQ <  − 40, ambidexterity with an EHI LQ between − 40 and 40 (− 40 ≤ EHI LQ ≤  + 40) and Right handedness with an EHI LQ >  + 40. The left-handed group and the right-handed group were matched for age and gender. Each group included 8 women and 7 men. The mean age was 27.2 years of age with a 2.8 SD in the right-handed group and 25.5 years of age with a 3.6 SD in the left-handed group. The right-handed group had a mean EHI Handedness LQ of 95.2 and the left-handed group a mean EHI Handedness LQ of − 93.

A medical history was recorded for every volunteer. Inclusion criteria was trilinguism with a different age acquisition of each language. The 3 spoken languages were English, French and Arabic.

Exclusion criteria included: ambidexterity, as well as neurologic, psychiatric, or any other significant medical disease.

We labelled L1, L2 and L3 the native language, the second and the last acquired language respectively. The mean age of acquisition was birth for L1, 3 years of age for L2 and 9 years of age for L3. The Arabic language represented L1 in 70% of the cases, the other 30% had the French language as a native language. L2 was the French language in 70% of our subjects, Arabic in 26.7% and English in 3.3% of our population.

The Common European Framework of Reference for Languages CEFRL was used as a questionnaire to objectively determine the language proficiency of the subjects. Their language habits including how many hours per day and in which context (social, professional) they use each language were also reported. Subjective proficiency was also assessed with the subjects ranking L1, L2 and L3 from the language they feel the most comfortable using (score of 1) to the language they feel the least comfortable using (score of 3). All of our subjects were highly proficient in all three languages.

The subjects demographics are reported in a previous article^29^ that focused mainly on the fMRI findings in this population of right and left-handed trilingual subjects.

### Image acquisition

Scanning was performed on a 3 T MRI scanner (GE Healthcare, Milwaukee, Wisconsin) using an eight-channel head coil. An axial FLAIR sequence (repetition time/echo time (TR/TE) = 10,000/140.2 ms, field of view (FOV) = 220 × 220 mm, flip angle = 90°, thickness = 4 mm) was performed to rule out parenchymal abnormalities. Three-dimensional axial T1-weighted images were acquired using a spoiled gradient-recalled echo sequence (TR/TE = 8.4/2.6 ms, FOV = 260 × 260 mm, flip angle = 15°, matrix = 256 × 256 voxels, thickness = 1.2 mm). fMRI data were then acquired using a single shot gradient echo echo-planar imaging (EPI) sequence: TR/TE = 3000/30 ms, FOV = 260 × 260 mm, flip angle = 90°, matrix = 128 × 128 voxels, thickness = 4.5 mm, no skip, EPI voxel size = 1.875 × 1.875 × 4.5 mm. A visual responsive naming paradigm^30^ was repeated 3 times, once for each language. The order of administration of L1, L2 and L3 paradigms was random and independent of the age of acquisition and language proficiency of the subject and it was counterbalanced across participants. A block design was used with a 30 s of activation (five questions) followed by a 30 s rest period where the subject was asked to fixate on a cross-hair. The block design was repeated five times (5 min in total). The blocks of activation (Epoch) were constructed similarly for each language and consisted of the same questions for each language. The order of the questions was however random within each block. The order of the blocks within each paradigm was also different to account for the effect of habituation^29^.

DTI scans were acquired using a whole-brain 34 direction diffusion-weighted images (DWI) with the following parameters: TR/TE = 8000/86 ms, FOV = 260 mm, matrix = 128 × 128 voxels, slice thickness = 2 mm, b values of 0 and 1000 s/mm^2^, voxel size = 2 × 2 × 2.6 mm.

### fMRI data analysis

Post processing and analysis of the fMRI images were performed using SPM12 available on the web: (https://www.fil.ion.ucl.ac.uk/spm/software/spm12/). The images were normalized into the International Consortium for Brain Mapping (ICBM) space template for European brains. Realignment of the functional, blood oxygenation level dependent (BOLD) images and 3D axial T1 weighted images was performed using the mean image as reference. In order to improve signal to noise ratio, realignment was then followed by smoothing using a Gaussian kernel of 8 mm full-width at half maximum.

We obtained statistical analyses of fMRI at the level of a single subject and then we performed another statistical analyses at a group level for both the left and the right-handed groups^29^.

### Language evaluation

The number of active clusters in the language areas and the number of active voxels (voxel size of 2 × 2 × 2 mm) in a cluster were reported by a neuroradiologist, blinded to the handedness of the subject, the age of acquisition and the language proficiency. The language areas that were studied included the activated clusters in the inferior frontal gyrus, the middle frontal gyrus, the superior frontal gyrus, the superior temporal gyrus, the middle temporal gyrus, the supramarginal gyrus and the angular gyrus^29^.

### Language lateralization by fMRI

The language laterality index (LI) was calculated for every subject for L1, L2 and L3 using the standard formula^31^ for fMRI: fMRI-LI = (*L* − *R*)/(*L* + *R*), where *L* and *R* are the numbers of voxels in the clusters of language in the left and right hemispheres, respectively. The fMRI-LI ranged from − 1 (complete right dominance) to + 1 (complete left dominance). Right hemisphere language laterality was defined as − 1 ≤ fMRI-LI <  − 0.2, bilaterality as − 0.2 ≤ fMRI-LI ≤ 0.2, and left hemisphere language laterality as 0.2 < fMRI-LI ≤ 1.

### DTI data analysis

**Figure 1:**
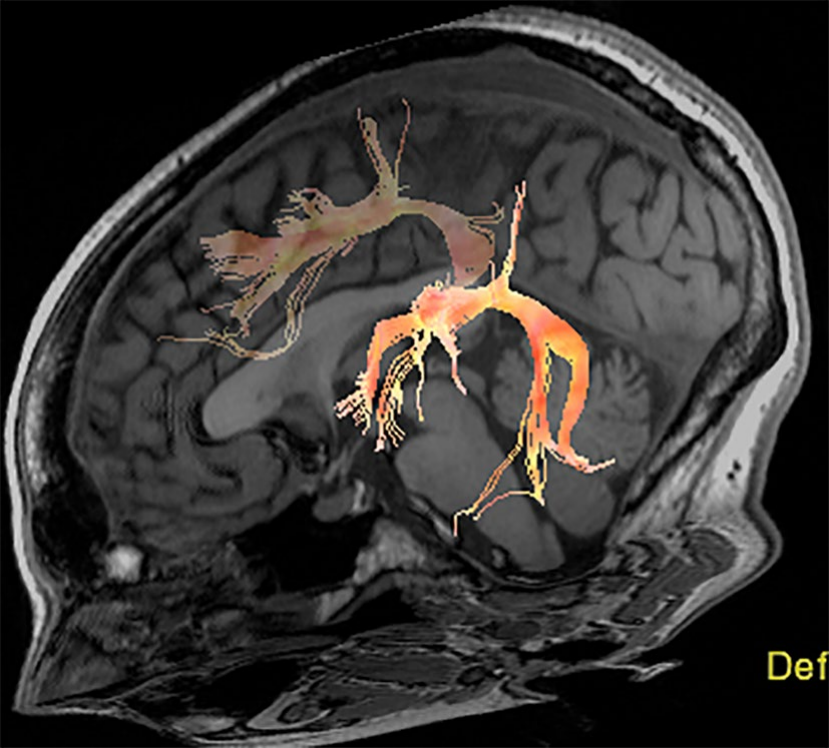
DTI of the direct pathway of the AF fused with anatomic T1 weighted sequence demonstrates the presence of a bilateral AF.

DTI images were reconstructed using the dcm2niigui application in MRIcron, a cross-platform NIfTI format image viewer (https://www.nitrc.org/projects/mricron). Alignment of the anatomical and DTI data for each subject was performed in AFNI software (https://afni.nimh.nih.gov) after gradient distortion correction and head motion correction. Images were then normalized into the International Consortium for Brain Mapping (ICBM) space template. The aligned and motion-corrected DTI data and diffusion gradients DTI were processed with the Diffusion Toolkit software developed by the Martinos Center for Biomedical Imaging, Massachusetts General Hospital (http://www.trackvis.org)^32^. For each voxel, a tensor matrix was derived. After diagonalization of the matrix, eigenvalues were obtained and maps of diffusivity (MD) and fractional anisotropy (FA) were created. Trackvis (http://www.trackvis.org/) was then used to reconstruct the AF in the left and in the right hemispheres using a region of interest (ROI) approach (Fig. 1). Deterministic tract reconstruction using a fiber association by continuous tracking algorithm (35-degree angular threshold) was performed for fiber-tracking analysis.

The images were processed by a neuroradiologist blinded to the patient’s handedness and to the results of the fMRI. Regions of interest for tract segmentation were placed manually on the color FA maps cross referenced to the b_0_ DWI images in the frontal and temporal lobes on the left and on the right, in the expected location of the AF. The AF was derived by a two-ROI approach, one at the level of the rolandic operculum in coronal view and the other laterally to the ventricular trigone on an axial view. Fibers that pass through both regions defined the AF^26^. Any aberrant fibers in reference to the known anatomy were removed. The AF in each subject was categorized as present on the left only, on the right only, or bilaterally. Mean Diffusivity (MD), mean FA, Number of Fibers, Fiber Length and Fiber Volume were then calculated for each identifiable AF.

### Language lateralization by DTI

The structural language laterality index for the AF determined by DTI (DTI-LI) was calculated for every subject using the standard DTI-LI formula: DTI-LI = (Left AFV − Right AFV)/(Left AFV + Right AFV), where AFV is the AF Volume. DTI-LI ≥  + 0.1 value indicates left hemispheric lateralization, ≤  − 0.1 indicates right hemispheric lateralization and the values between + 0.1 and − 0.1 represent bilateral lateralization^12,14,17^.

### Comparison of DTI-LI and fMRI-LI

The first language acquired being the native language of the subject, language lateralization on fMRI for the first language acquired L1 (LI-L1) was adopted as the language lateralization of the subject on fMRI. A prior fMRI study conducted on these patients demonstrated that irrespective of language proficiency and age of acquisition, the lateralization of language does not change for right-handed subjects. It does however change for some left-handed subjects, with the language of the least proficiency (usually L2 or L3) presenting a bilateral activation in few cases^29^.

Statistical analysis was performed using the Statistical Package for Social Sciences (SPSSv23). Correlation between FA Index (Left FA − Right FA)/(Left FA + Right FA), DTI-LI and fMRI Laterality Index (LI-L1) was determined using Pearson correlation. Comparison between the left AF and the right AF regarding MD, FA, Number of Fibers, Fiber Length, Fiber Volume was evaluated using Student’s t-test and the ANOVA test. The Independent Sample t test and the ANOVA test were used to determine if there were differences between the AF of the right-handed subjects and the AF of the left-handed subjects in the right hemisphere and in the left hemisphere. The statistical significance threshold was set to *p* < 0.01, adjusted for multiple comparisons with Bonferroni correction.

## Results

17 of the 30 participants had a normal brain MRI. 13 of the 30 subjects had minor brain abnormalities: 7 participants presented nonspecific signal abnormalities of the white matter, 5 had pineal cysts, and one subject demonstrated an arachnoid cyst. None of these abnormalities was significant enough to interfere with the results of the DTI or the fMRI.

One left-handed subject had a DTI sequence that was degraded due to motion artifacts and was excluded from the study.

27 subjects presented a bilateral AF. We were not able to trace the AF in both hemispheres for two of the right-handed volunteers. For one of them, we were only capable to trace a left AF and for the other a right AF. Both subjects demonstrated a left lateralization of language on the fMRI. The inability to track the AF bilaterally for some subjects has already been described in previous studies^10,33^.

**Figure 2:**
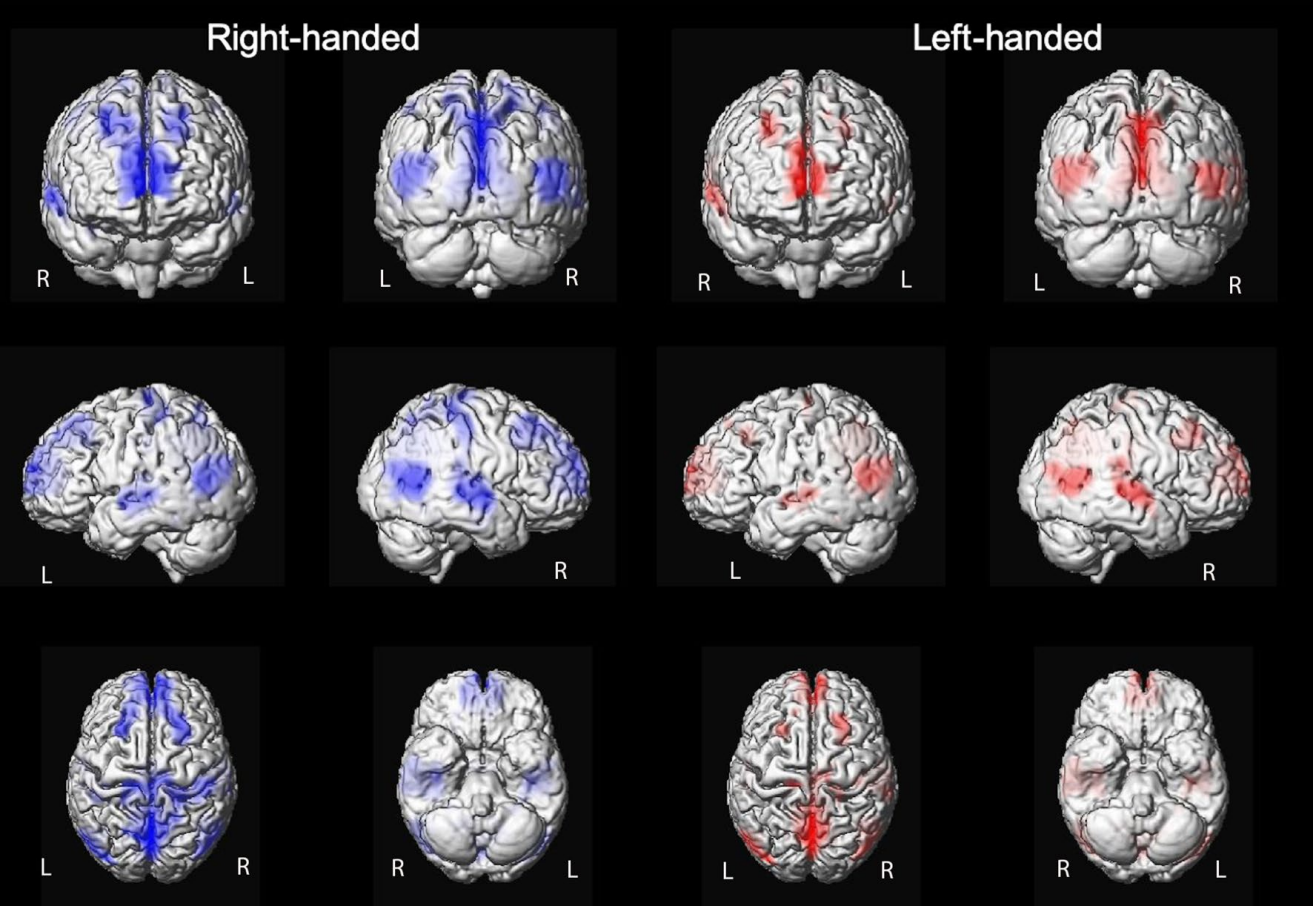
Group maps activation on fMRI for both right-handed and left-handed subjects demonstrate a bilateral cortical activation with a major activation in the right hemisphere as well as the left hemisphere.

On fMRI and at a group level, there was left hemispheric and right hemispheric activation in both the right-handed and left-handed groups with high activation in both hemispheres (Fig. 2). 15 out of 29 subjects (52%) were found to have a bilateral lateralization of the AF on DTI. They were equally distributed between the right-handed and the left-handed groups, Chi-Square *p* = 0.766 (Table 1). The left-handed subjects demonstrating bilateral lateralization and right lateralization of language on fMRI presented corresponding lateralization of the AF on DTI. The subject presenting right lateralization of language on fMRI but left lateralization of AF on DTI was the only right-handed subject presenting right dominance of language. For the left-handed and right-handed subjects with left lateralization of language on fMRI, the laterality of the AF on DTI was variable (50% of the cases bilateral, 25% on the right and 25% on the left). Only 4 subjects had bilateral lateralization of language on fMRI and they were all left-handed (Table 2). Pearson’s correlation was run to determine the relation between characteristics of the AF on DTI, DTI-LI, FA Index and fMRI-LI. There was no correlation between DTI-LI and fMRI-LI, neither in the right-handed group (r = 0.01, *p* = 0.973) nor in the left-handed one (r = 0.137, *p* = 0.64). Similarly, no correlation was found between the FA Index and fMRI-LI, neither in the right-handed group (r =  − 0.255, *p* = 0.36) nor in the left-handed one (r = 0.049, *p* = 0.868). There was no relationship or association between the DTI-LI and the fMRI-LI in the total group of the participants (*p* = 0.766). For the right AF of the right-handed subjects, there was a positive correlation between the FA and the fMRI-LI (r = 0.545, *p* = 0.04), and a negative correlation between the Fiber Volume and the fMRI-LI (r =  − 0.614 *p* = 0.02) as well as between the Number of Fibers and the fMRI-LI (r =  − 0.565, *p* = 0.035). These findings did not however survive multiple comparisons correction. In the left-handed subjects, there was no correlation between the DTI characteristics of the right and left AF and the fMRI-LI (Table 3). The MD was significantly different between the right AF and the left AF in the global population (*p* < 0.001), in the right-handed group (*p* = 0.017) and in the left-handed group (*p* = 0.002), with a lower MD of the right AF. The FA, the Volume and Length were not significantly different between the right AF and the left AF in the total group of participants, the left-handed and the right-handed groups (Table 4). Using the Independent Samples t-Test, there was no difference between the right-handed group and the left-handed group regarding the FA, Fiber Volume, Number of Fibers, MD and Length of the AF. The ANOVA test confirmed the results of the Independent Samples t-Test and demonstrated that the only significant difference in the characteristics of the right AF and the left AF in each of the two groups was the MD with a lower MD of the right AF compared to the left (*p* = 0.02 in the left-handed group and *p* = 0.017 in the right-handed group). The ANOVA test did not demonstrate a significant difference in the characteristics of the right AF between the right and the left-handed groups. There was as well no significant difference in the characteristics of the left AF between the right and the left-handed groups.

**Table 1:** Laterality of the AF on DTI in relation to handedness of the subject.

	Laterality of AF on DTI			Total
	Right	Bilateral	Left	
**Handedness**				
Right-handed	5	7	3	15
Left-handed	3	8	3	14
Total	8	15	6	29

**Table 2:** Laterality of language on fMRI and on DTI.

Number of subjects	Laterality of AF on DTI			Total
	Right	Bilateral	Left	
**Laterality of language on fMRI**				
Right	2	0	1	3
Bilateral	0	4	0	4
Left	6	11	5	22
Total	8	15	6	29

**Table 3:** Pearson correlation test between numerous DTI variables and fMRI-LI regarding handedness.

		fMRI-LI	
		Right-handed	Left-handed
**Right AF**			
Fractional anisotropy	Correlation coefficient	0.545*	0.391
	Sig. (2-tailed)	0.044	0.167
Volume	Correlation coefficient	− 0.614*	0.335
	Sig. (2-tailed)	0.020	0.242
Number of fibers	Correlation coefficient	− 0.565*	0.330
	Sig. (2-tailed)	0.035	0.250
Mean diffusivity	Correlation coefficient	− 0.340	− 0.240
	Sig. (2-tailed)	0.234	0.408
Length	Correlation coefficient	− 0.196	0.226
	Sig. (2-tailed)	0.501	0.438
**Left AF**			
Fractional anisotropy	Correlation coefficient	0.020	0.248
	Sig. (2-tailed)	0.945	0.392
Volume	Correlation coefficient	− 0.081	0.375
	Sig. (2-tailed)	0.784	0.186
Number of fibers	Correlation coefficient	− 0.068	0.350
	Sig. (2-tailed)	0.817	0.220
Mean diffusivity	Correlation coefficient	− 0.096	− 0.295
	Sig. (2-tailed)	0.743	0.306
Length	Correlation coefficient	0.086	− 0.093
	Sig. (2-tailed)	0.771	0.752
DTI-LI	Correlation coefficient	0.010	0.137
	Sig. (2-tailed)	0.973	0.640
FA index	Correlation coefficient	− 0.255	0.049
	Sig. (2-tailed)	0.360	0.868

*Corresponding *p* < 0.05.

**Table 4:** Mean characteristics of the AF on DTI regarding handedness.

	Right-handed group			Left-handed group		
	Left hemisphere	Right hemisphere	*p* value	Left hemisphere	Right hemisphere	*p* value
Fractional anisotropy	0.389	0.382	0.726	0.377	0.415	0.140
Volume (cm^3^)	16.43	16.47	0.984	15.63	15.42	0.881
Number of fibers	1455.07	1448.36	0.970	1488.00	1428.79	0.737
Mean diffusivity	1193.89	1023.63	0.017*	1176.18	995.17	0.002*
Length (cm)	28.89	28.48	0.773	27.84	26.75	0.372

*p < 0.01 after Bonferroni correction.

## Discussion

This study utilized DTI to examine the hemispheric asymmetry of the AF in healthy trilingual subjects in order to determine if the language lateralization on fMRI correlates with the asymmetry of the AF and whether this relation varies with the handedness of the subjects. We hypothesized that most subjects would present leftward AF asymmetry and that the lateralization of the AF on DTI would correlate with the lateralization of language by fMRI, at least in the right-handed subjects who in 92.5% demonstrate typical left hemisphere dominance of language^4^.

Our 4 main findings are: (1) The absence of significant asymmetry of the AF on DTI; (2) a lower diffusivity of the right AF compared to the left one; and (3) no correlation between the DTI-LI and fMRI-LI, the only subjects who presented corresponding DTI-LI and fMRI-LI were the left-handed subjects with atypical bilateral or right lateralization of language on fMRI.

The absence of asymmetry of the AF is an unexpected finding that has not been reported before. On the contrary, many authors reported leftward asymmetry of the AF. Powell et al.^16^ demonstrated greater Volume and FA in the left AF compared to the right in 10 right-handed native English-speaking healthy subjects. Sreedharan et al.^17^ reported larger Volume of the left AF compared to the right in ten healthy right-handed monolingual children. This was corroborated by Propper et al.^12^ who found that overall the right hemisphere’s AF volume is 41% smaller than the left hemisphere’s, a finding similar to those of Hagmann et al.^34^ who, furthermore, reported a decreased asymmetry of the AF in non-right-handed men and in women. Asymmetry in the relative fiber density (ratio of the number of arcuate tracts to the total number of fiber tracts generated in the arcuate ROI) has also been reported by Nucifora et al.^15^ with a greater relative fiber density in the left AF of 27 right-handed subjects, and by Vernooij et al.^14^ who also showed a leftward asymmetry in relative fiber density of the AF that was irrespective of handedness or functional language lateralization. Asymmetry in the FA with greater FA values of the left AF were reported by Rodrigo et al.^35^, and Allendorfer et al.^36^ who also found that AF asymmetry is independent from hand preference. Greater FA values, Volume and Number of Fibers of the AF were present in the dominant hemisphere for language in 10 patients with epilepsy studied by Delgado et al.^37^ who compared DTI results to lateralization of language by WADA testing. In our study, and irrespective of the handedness of the subjects, there was no asymmetry of the AF regarding the FA, Volume, Length and Number of fibers. This discrepancy in the results might be explained by the fact that the subjects involved in our study were trilingual subjects. Multilingualism has been determined to have a major role in the reorganization of the brain white matter. It affects the structural organization of the language pathways. It increases FA values and leads to greater white matter integrity^26,38,39^. A single longitudinal study^26,27^ has compared FA values between groups of mono- and bilingual children. The authors reported increased FA values for simultaneous bilinguals only along the left inferior fronto-occipital fasciculus, but found no effects of bilingualism on the AF in these child participants. Another study conducted by Hämäläinen et al.^40^ had contradictory results and found a more prominent role of the right AF in multilingual subjects leading to a more bilateral organization of the perisylvian language-related tracts. It also showed that the late bilingual speakers are more likely to exhibit extreme left lateralization pattern than the early bilinguals suggesting that early-onset bilingualism contributes to a more bilaterally balanced structural configuration of the perisylvian language tracts. Catani et al.^33^ have also found that a higher symmetry in the structure of the AF predicted better performance in verbal recall task. As bilinguals tend to exhibit more bilateral functional activations in linguistic tasks^22^, this is probably reflected in the underlying bilateral structural organization of the AF. In a previous study involving the same population of trilingual subjects^29^, it was demonstrated that the functional group maps activation of each of the three spoken languages, in the right-handed and left-handed groups, demonstrated a bilateral language activation with a prominent activation seen in the right hemisphere as well as in the left hemisphere. This pronounced cortical activation in the right hemisphere confirms the theory of Hull and Vaid^22^ and the findings of Polczynska et al.^41^ of a more pronounced right hemisphere activation with early bilingualism. This larger role of the right hemisphere in language activation in early bilingual subjects might also explain a more important role of the white matter involved in language in the right hemisphere, hence a more balanced bilateral organization of the AF on DTI in early bilingual and multilingual subjects.

The only significative difference that we found between the right and left AF was a lower MD of the right AF compared to the left, irrespective of handedness*.* This finding contradicts the results of Rodrigo et al.^35^ who demonstrated leftward asymmetry of the AF in 18 right handed subjects with a higher FA in the left AF compared to the right but no difference in MD. The overall diffusivity and the degree of directionality of diffusion in a tissue can be quantified by MD and FA, respectively^42^. Directional diffusivities such as axial diffusivity (diffusivity along the axon) and radial diffusivity (diffusivity perpendicular to the axon) are more specific to underlying biological processes, such as myelin and axonal changes and increased radial diffusivity values for instance have been linked to de- or dysmyelination of axons^43^. The relationship between the radial diffusivity, the axial diffusivity, the FA and the MD, is such that FA increases when radial diffusivity decreases and/or axial diffusivity increases while MD increases when axial diffusivity and/or radial diffusivity increases^43^. MD has been described to be an inverse measure of the membrane density^44^. Decreased MD in particular with increased FA has been suggested to reflect increasingly dense and ordered packing of the fiber tracts^45,46^. Therefore, the decreased MD of the right AF independently of the handedness of the subjects reflects a denser axonal packing and higher fiber organization in the right AF in multilingual subjects. This is in line with Hämäläinen et al.^40^ who have found significantly lower MD along the right posterior segment of the AF in early bilinguals compared to late bilinguals. These findings also support the hypothesis of a more important role of the right hemisphere and the right AF in multilinguism.

While several studies have shown concordance between the lateralization on the AF on DTI and the lateralization of language on fMRI^12,17,36,47,48^ and concordance of the LI-DTI with language lateralization on WADA testing^37^, we didn’t find any correlation between the LI-fMRI and the lateralization of language on DTI. In prior studies, Vassal et al.^48^ demonstrated a leftward asymmetry of Volume of the AF that correlated with the lateralization of language on fMRI in 20 healthy monolingual right-handed subjects, Silva et al.^47^ also showed a correlation of the AF volume asymmetry with fMRI in right-handed healthy subjects, and while Vernooji et al.^14^ showed that functional and structural asymmetries correlated in the right but not in the left-handers, Allendorefer et al.^36^ demonstrated that AF asymmetry was in relation to language function but not to handedness. Many studies, in children^17^ and in adults^37^ concluded that the DTI lateralization of the AF could replace evaluation of the lateralization of language by fMRI or WADA testing respectively in monolingual subjects. The novelty of this study relies on the fact that it involves trilingual subjects and compares the results of the DTI of the AF to the fMRI activation of language in a multilingual population. The relation between the fMRI activation of language and the organization of the AF has not been described before in a multilingual population. This study also involves a relatively large number of left-handed subjects, and permits evaluation of the organization of the AF in multilinguals in relation to handedness. Our conflicting results with the literature^12,15–17,34^, including the absence of significant asymmetry of the AF on DTI and the absence of correlation between the DTI-LI and fMRI-LI, might be explained by the fact that our population is trilingual and that multilinguism leads to a balanced bilateral reorganization of the white matter of the AF. With a bilateral reorganization of the AF, the DTI-LI does not correlate anymore with the degree of cortical activation and lateralization on fMRI. The only subjects who had corresponding DTI-LI and LI-fMRI were the left-handers with an atypical bilateral or right lateralization of language on fMRI. In view of the absence of correlation between the DTI-LI and the lateralization of language on fMRI, it is safe to conclude that DTI is not able to predict language lateralization in early multilingual highly educated subjects.

This study has some limitations. First, this study does not include a large sample size or a monolingual comparison group. This limitation might raise the concern of the reproducibility of tracking the AF tract by a ROI-ROI approach and whether there might have been a variability in defining the AF tract and its FA, MD, Volume and length values between the subjects. This concern does not seem to be valid as the white-matter deterministic fiber tracking method and the software adopted in this study to delineate the AF were used by Allendorfer et al.^36^ to demonstrate an asymmetry of the AF in their monolingual population. Furthermore, Paldino et al.^49^ proved the repeatability of the quantitative metrics derived from MR diffusion tractography using the same Diffusion Toolkit and Trackvis software in pediatric patients with epilepsy. The second limitation of this study is the fact that all the subjects were high functional post-doctoral adults, who had high proficiency in all 3 languages and used the 3 languages daily in their professional and social lives. Our subjects had an early age of acquisition for the 3 languages, with a mean age of acquisition of the last language acquired (L3) around 9 years of age. The early age of acquisition of L2 and L3 and the high proficiency might have led to a bilateral organization of language. It is therefore difficult to extrapolate the results of our study to all multilingual subjects who might be less proficient in their second language and who might have a late age of second language acquisition. Several studies have demonstrated that the increase in FA varies with the age of acquisition of the second language and whether the subjects are sequential or simultaneous bilinguals^27,39,40^. Finally, in this study we only reviewed the characteristics of the arcuate fasciculi. Since there are other regions involved in language processing and other white matter tracts affected by bilingualism^39^, a more expanded language network needs to be evaluated and further studies will be a necessary adjunct to future investigation beyond the AF. Additional studies with larger sample size, comparing the DTI characteristics of a matched monolingual population and a bilingual population and their relation to language lateralization on fMRI are also required to ascertain the role of bilingualism on the bilateral white matter organization of language.

## Conclusion

While in monolingual subjects, the leftward asymmetry of the volume of the AF is correlated to a left lateralization of language on fMRI, this study demonstrates a tendency to a more pronounced role of the right hemisphere and right AF in trilingual subjects. In our multilingual population, independent of handedness, there is no correlation between the FA Index, the volume laterality of the AF on DTI and the lateralization of language on fMRI. In this highly educated early trilingual population, there is a more balanced bilateral lateralization of the AF on DTI. These findings suggest the idea that bilingualism and multilingualism lead to a more bilaterally balanced structural organization of the AF making it difficult to predict the lateralization of language in multilingual subjects based solely on DTI.
